# Diabetes distress and disordered eating behaviors in youth with type 1 diabetes: the mediating role of self-regulatory fatigue and the moderating role of resilience

**DOI:** 10.1186/s40337-023-00838-4

**Published:** 2023-07-22

**Authors:** Meijing Zhou, Hong Wang, Jian Yu, Dan Luo, Min Zhu, Mei Zhang, Jingjing Xu, Tao Yang

**Affiliations:** 1 Department of Endocrinology, The First Affiliated Hospital of Nanjing Medical University, 300 Guangzhou Road, Nanjing, 210029 China; 2 School of Nursing, Nanjing University of Chinese Medicine, 138 Xianlin Avenue, Nanjing, 210029 China; 3 Department of Nursing, The First Affiliated Hospital of Nanjing Medical University, 300 Guangzhou Road, Nanjing, 210029 China

**Keywords:** Type 1 diabetes, Disordered eating behaviors, Self-regulatory fatigue, Resilience, Diabetes distress

## Abstract

**Background:**

Despite previous research on the association between diabetes distress and disordered eating behaviors (DEBs) among youth with type 1 diabetes (T1D), there is a lack of understanding regarding the underlying mechanisms. This study aimed to investigate the relationships between diabetes distress and DEBs, specifically examining whether self-regulatory fatigue mediated the relationship and whether resilience moderated this mediation.

**Methods:**

A cross-sectional study was performed among youth with T1D recruited from two diabetes centers in Nanjing, China. Measurement instruments included the problem areas in the diabetes-5 scale, the diabetes strengths and resilience measure for adolescents, the self-regulatory fatigue scale, and the Chinese version of diabetes eating problem survey-revised. Mediation and moderated mediation analyses were conducted.

**Results:**

A total of 185 youths with T1D were involved in the current study. The results indicated that diabetes distress positively predicted DEBs. Self-regulatory fatigue partially mediated the association between diabetes distress and DEBs, accounting for 50.88% of the overall effect. Additionally, the pathway from self-regulatory fatigue to DEBs was moderated by resilience.

**Conclusion:**

The current study examined whether self-regulatory fatigue mediated the relationship between diabetes distress and DEBs and whether resilience moderated the connection between self-regulatory fatigue and DEBs. These findings add to the theoretical basis of how diabetes distress influences DEBs and help guide the incorporation of diabetes distress, self-regulatory fatigue, and resilience into DEBs reduction programs for youth with T1D.

## Abstract (plain language summary)

A high prevalence of disordered eating behaviors (DEBs) has been observed among youth with type 1 diabetes (T1D), leading to poor glycemic control, significant short- and long-term consequences, and an increased mortality risk. It is crucial to closely monitor DEBs in this population and identify factors that can be modified to develop targeted interventions. While previous studies have shown that diabetes distress, which refers to negative emotions specific to diabetes, is a positive predictor of DEBs, no research has examined how diabetes distress affects DEBs. Our cross-sectional study discovered a significant association between diabetes distress, self-regulatory fatigue, and an increased likelihood of experiencing DEBs. Furthermore, low resilience exacerbates this relationship, whereas high resilience weakens it. These findings highlight the importance of integrating resilience, self-regulatory fatigue, and diabetes distress in DEBs reduction programs among youth with T1D.

## Background

Type 1 diabetes (T1D) is primarily an immune-mediated disorder characterized by a deficit or absence of endogenous insulin due to destroying pancreas beta cells [1]. This condition predominantly affects adolescents and young adults, and T1D patients need lifelong self-management to achieve optimal glycemic control and prevent complications [2]. Daily self-management of T1D involves carefully balancing exogenous insulin supply with food intake and exercise, as well as regular blood glucose checking [1]. The intense focus on food in connection with blood glucose regulation and the dietary restrictions may lead to disordered eating behaviors (DEBs) in T1D [3]. DEBs refer to abnormal eating attitudes and behaviors, including unhealthy weight-control practices, excessive exercise, self-induced vomiting, dietary restraint, diet pills, abuse of laxatives, and breaking dietary rules [3]. Notably, one manifestation of DEBs in T1D is intentional insulin omission, which enables weight loss without food restriction [4]. Patients reporting insulin omission have higher HbA1c levels and are at higher risk of complications such as retinopathy and nephropathy [5, 6].

Diabetes distress is a negative emotion stemming from the challenges of living with diabetes and self-management demands [7]. An Australian national study indicated that 54% of adolescents with T1D reported medium to severe diabetes distress [8]. High levels of diabetes distress have been associated with elevated average blood glucose levels, higher HbA1c values, and lower time in range (a suboptimal glycemic control metric derived from continuous glucose monitoring data) [9, 10]. Several studies have verified that diabetes distress is a positive predictor of DEBs [11–13]. However, the mechanism by which diabetes distress influences DEBs is still unclear.

### Self-regulatory fatigue as a mediator

Suffering from T1D can be considered a chronic stressor, and individuals’ ability to regulate their thoughts, feelings, and behavior determines how they react to stressors. Notably, the capacity to self-regulate is a limited psychological resource that can be depleted [14]. When significant self-regulatory effort is required for a particular task, there is less capacity available for subsequent tasks, leading to a state known as self-regulatory fatigue [14]. According to the self-control strength model, prolonged efforts to control negative emotions can result in self-regulatory fatigue, which can subsequently bring about self-control failures, such as engaging in alcohol abuse, binge eating, or aggressive behaviors [15, 16]. Studies outside the field of T1D have shown that self-regulatory fatigue is significantly associated with psychological distress [17, 18]. Moreover, a study confirmed that self-regulatory fatigue could cause impulse intensification and emotional dysregulation [19], which may predispose individuals to DEBs [20, 21]. Therefore, it is plausible to speculate that self-regulatory fatigue may mediate the relationship between diabetes distress and DEBs.

### Resilience as a moderator

Although diabetes distress may impact DEBs through self-regulatory fatigue, not all youth with T1D are equally sensitive to this effect. Resilience may play an important role in buffering the impact [22, 23]. In this study, resilience is defined as an individual’s quantifiable and modifiable ability to thrive despite adversity [24]. Based on Rutter’s resilience development model, resilient individuals can effectively use their internal strength and external environmental resources to buffer adverse chain reactions caused by risk factors [25]. One such risk factor is self-regulatory fatigue linked to DEBs. Additionally, studies proved that higher resilience contributed to regulating emotions and self-control, potentially reducing the impact of self-regulatory fatigue on DEBs [26, 27]. Moreover, Boselie et al. found that optimism, a significant component of resilience, can buffer the effects of self-regulatory fatigue on executive task performance [22]. Accordingly, we could deduce that resilience moderates the association between self-regulatory fatigue and DEBs.

### Present study

Understanding the pathway from diabetes distress to DEBs contributes to advancing prevention and intervention efforts targeting DEBs. To address the current literature gaps, we performed a cross-sectional study to examine the associations among diabetes distress, self-regulatory fatigue, resilience, and DEBs. We hypothesized that (1) self-regulatory fatigue would mediate the relationship between diabetes distress and DEBs in youth with T1D and (2) resilience might buffer the impact of self-regulatory fatigue on DEBs.

## Methods

### Participants

Convenience sampling was used to enroll participants from two national pediatric diabetes centers in Nanjing, China, between December 2021 and September 2022. This study included youth with T1D aged 10–24 years, diagnosed for more than six months, and able to comprehend and answer the survey questionnaires in Chinese. We excluded those with concurrent psychiatric conditions, malignant tumors, or other chronic diseases (such as asthma and arthritis), as determined by their medical charts. Participants diagnosed with eating disorders based on medical charts were also ineligible for this study. Youths who scored ≥ 20 on the Diabetes Eating Problem Survey-Revised (DEPS-R) underwent evaluation by a psychiatrist to assess for potential eating disorders, and those diagnosed with eating disorders were subsequently excluded from the study. The current study was carried out in conformity with the Declaration of Helsinki. We adopted PASS 15.0.5 software to calculate the sample size. The regression model type was unconditional, with 11 independent variables (diabetes distress, self-regulatory fatigue, resilience, demographic, and clinical variables). The minimum required sample size was 181 to maintain a statistical power of 0.8, an effect size (between small and medium) of 0.10, and a significance threshold (α) of 0.05. We expanded the sample size to 202 after accounting for invalid responses. Finally, 185 participants’ data were used for statistical analysis because 17 had missing data.

### Measures

#### Diabetes distress

Diabetes distress was measured by the Problem Areas in Diabetes (PAID)-5 scale, a validated and reliable short form of the full 20-item PAID scale [28]. Each item has five possible response options, from “not a problem” (0 points) to “a serious problem” (4 points). The sum score of this scale ranges from 0–20, with a higher score indicating more severe diabetes distress. The PAID-5 scale was previously widely used in Chinese youths (8–24 years old) and showed excellent reliability and validity [29]. The Cronbach’s α of the PAID-5 scale in this study was 0.893.

### Resilience

We used a validated Chinese version of the diabetes strengths and resilience measure for adolescents (DSTAR-Teen) to measure the resilience of youth with T1D [30]. DSTAR-Teen was initially developed by Hilliard et al. and revised by Xu et al. [31]. This scale contains 12 items, comprising three dimensions: help-seeking, diabetes-related confidence, and family resources. Each item is scored with a five-point Likert scale (1 = never, 2 = rarely, 3 = sometimes, 4 = often, 5 = almost always), with a total score between 12 and 60 points. The higher the score, the stronger the resilience. DSTAR-Teen was adopted to explore resilience among Chinese youths with T1D aged 8–24 years old, and good psychometric properties of this scale were found [31]. The Cronbach’s α in our sample was 0.885.

### Self-regulatory fatigue

The self-regulatory fatigue scale (SRF-S), designed by Nes [32] et al. and translated to Chinese by Wang et al. [33], was adopted to evaluate the level of chronic self-regulatory fatigue. The SRF-S includes 16 items and involves three dimensions, cognition, emotion, and behavior. Responses to each item are graded from “totally disagree” to “absolutely agree” on a scale of 1 to 5, with a total score that ranges from 16 to 80. A higher score reflects a greater level of self-regulatory fatigue. This scale was reliable and valid in Chinese‐speaking populations with chronic illnesses [34]. The Cronbach’s α of SRF-S in this study was 0.752. To further verify the construct validity of SRF-S, we performed the confirmatory factor analysis with the same sample. The acceptable model fit indexes were found: c^2^/df = 2.012, GFI = 0.883, IFI = 0.880, CFI = 0.876, RMSEA = 0.074.

### Disordered eating behaviors

We used a validated Chinese version of DEPS-R to identify DEBs among youth with T1D. The original scale was developed by Antisdel et al. [35] and then revised by Markowitz et al. [36]. The DEPS-R includes 16 items, each being answered from “never” to “always” and scored with 0–5 points. The sum scores range between 0 and 80, with scores ≥ 20 indicating the presence of DEBs, warranting further clinical evaluation. Good psychometric properties of DEPS-R were found in Chinese youths (8–17 years old) and adults (≥ 18 years old) with T1D [37]. The Cronbach’s α in this sample was 0.808.

### Procedures

The First Affiliated Hospital Ethics Committee of Nanjing Medical University approved the study (2021-NT-49). Written informed consent was obtained from all participants before they were recruited into our study. For patients under 18, we have also gotten their parents’ consent. Before formally conducting the survey, we carried out a pre-survey to ensure that all youths could understand the questionnaire items and be able to fill them out independently. Five survey parts were delivered using structured paper and pencil questionnaires: sociodemographic (age, sex, education level, residence, family monthly income) and disease-related information (diabetes duration, insulin regimen), the PAID-5 scale, the DSTAR-Teen, the SRF-S, and the DEPS-R. Participants filled out these questionnaires in a quiet room at each clinical center. Investigators were responsible for answering all participants’ queries about the questionnaires. All questionnaires were immediately collected and reviewed after an average completion time of 20 min. Notably, when participants aged 10–13 completed the questionnaire, investigators randomly selected five items to check whether they fully understood them. If any misunderstanding existed, investigators and the patient would check all items in the questionnaires one by one to ensure the quality of the filling. Notably, none of the patients knew in advance that their questionnaires would be checked. After completing the questionnaire, all participants’ body weight and height were measured using electronic scales and a stadiometer. BMI was calculated by dividing the body mass in kilograms by the height in meters squared.

### Data analysis

The data were double-entered using EpiData 3.1 software, and all analyses were conducted using SPSS 22.0 software. Prior to analysis, the normality of variables was assessed by examining skewness and kurtosis values. Descriptive analysis, independent t-tests, and ANOVA were performed to describe the characteristics and distribution of DEBs. Sociodemographic and clinical factors that showed statistical significance with DEBs were included as control variables in the mediation and moderated mediation models. Bivariate correlations were explored among diabetes distress, self-regulatory fatigue, resilience, and DEBs. Hierarchical multiple regression models were employed to investigate whether self-regulatory fatigue mediated the relationship between diabetes distress and DEBs, and whether resilience moderated the association between self-regulatory fatigue and DEBs. The PROCESS macro in SPSS 22.0 (Model 4) was utilized to test the indirect effect of diabetes distress on DEBs [38]. Additionally, the PROCESS macro in SPSS 22.0 (Model 14) was adopted to validate the moderating effect of resilience on the pathway from diabetes distress to self-regulatory fatigue to DEBs [38]. A simple slope analysis was conducted to analyze the moderating effect of resilience further. A total of 5,000 bootstrap samples were used for percentile confidence intervals. Statistical significance was indicated by the absence of a zero in the confidence intervals.

## Results

### Descriptive analyses

The study enrolled a total of 185 youths with T1D. Table 1 provides an overview of their sociodemographic and clinical characteristics and the distribution of DEBs. Among the participants, 49.19% were under 18, and 57.80% were female. Overweight or obese patients accounted for 14.60% of the sample. In terms of residence, 51.35% of participants lived in urban areas, while only 31.35% had a monthly family income of 10,000 yuan or higher. Regarding disease duration, 43.24% of participants had been diagnosed with T1D for over five years. Moreover, 47.57% of patients used insulin pens for blood glucose control. DEBs did not show significant differences based on participants’ age, education, family monthly income, diabetes duration, or insulin regimen. However, significant differences were observed in sex, BMI, and residence (all *p* < 0.05).

**Table 1:** Sociodemographic and clinical characteristics of the participants and the distributions of disordered eating behaviors in categorical items (N = 185)

Characteristics	N (%)	Disordered eating behaviors		
		Mean (SD)	*F/t*	*p*-value
*Sex*			− 3.883	0.000
Male	79 (42.70)	18.38 (9.37)		
Female	106 (57.80)	23.99 (9.97)		
*Age*			− 0.832	0.407
<18	91 (49.19)	20.97 (10.66)		
≥ 18	94 (50.81)	22.20 (9.52)		
*BMI classification*			4.116	0.018
Underweight	23 (12.43)	21.61 (8.96)		
Normal	135 (72.97)	20.59 (10.18)		
Overweight or obese	27 (14.60)	26.59 (9.34)		
*Education*			0.417	0.660
Primary education	13 (7.03)	20.69 (7.43)		
Secondary education	81 (43.78)	22.36 (11.51)		
Higher education	91 (49.19)	21.04 (9.04)		
*Residence*			5.494	0.005
City	95 (51.35)	19.26 (9.72)		
Town	51 (27.57)	23.86 (9.79)		
Countryside	39 (21.08)	24.31 (10.24)		
*Family Monthly income (yuan)*			0.740	0.530
<3000	13 (7.03)	22.62 (9.51)		
3000–5000	42 (22.70)	22.69 (9.67)		
5–10,000	72 (38.92)	22.06 (9.98)		
>10,000	58 (31.35)	20.00 (10.68)		
*Diabetes duration (year)*			1.588	0.194
0.5–1	28 (15.14)	20.71 (8.70)		
1–3	43 (23.24)	19.07 (10.97)		
3–5	34 (18.38)	23.53 (10.41)		
>5	80 (43.24)	22.44 (9.78)		
*Insulin regimen*			− 0.170	0.865
Insulin pump	97 (52.43)	21.47 (9.94)		
Insulin pen	88 (47.57)	21.73 (10.30)		

For participants aged 18 years or older, their BMI was classified into three groups, namely underweight, normal weight, and overweight or obesity, based on the Chinese adult overweight and obesity prevention and control guideline. For participants under 18 years old, their BMI was categorized into three groups (underweight/normal weight/overweight or obesity) based on sex and age, using the Chinese screening standard for malnutrition, overweight, and obesity among school-age children and adolescents

Table 2 shows the means, standard deviation (SD), and Pearson correlations for DEBs, diabetes distress, self-regulatory fatigue, and resilience. Pearson correlation analyses indicated that DEBs were positively related to diabetes distress (*r* = 0.508, *p*<0.01) and self-regulatory fatigue (*r* = 0.661, *p*<0.01). Diabetes distress was positively associated with self-regulatory fatigue (*r* = 0.489, *p*<0.01). Resilience was negatively correlated with self-regulatory fatigue (*r* = − 0.619, *p*<0.01) and DEBs (*r* = − 0.516, *p*<0.01).

**Table 2:** Correlations between study variables

Variable	Range	Mean (SD)	1	2	3	4
1. Diabetes distress	0–20	13.09 (4.80)	1			
2. Self-regulatory fatigue	0–80	44.39 (7.13)	0.489**	1		
3. Resilience	0–60	37.19 (9.04)	− 0.420**	− 0.619**	1	
4. Disordered eating behaviors	0–80	21.59 (10.09)	0.508**	0.661**	− 0.516**	1

**p < 0.01

### Testing for mediation model

**Fig. 1:**
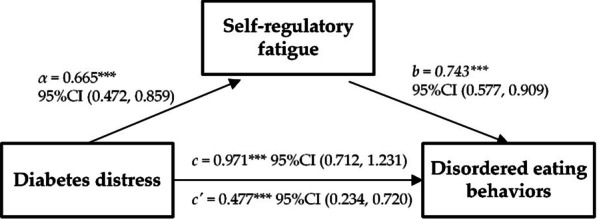
The mediation model of diabetes distress, self-regulatory fatigue, and disordered eating behaviors. **α** The effect of diabetes distress on self-regulatory fatigue; **b** The effect of self-regulatory fatigue on disordered eating behaviors; **c** The total effect of diabetes distress on disordered eating behaviors; **c’** The direct effect of diabetes distress on disordered eating behaviors; ***p < 0.001. Unstandardized beta coefficients were reported.

Whether self-regulatory fatigue mediates the relationship between diabetes distress and DEBs in youth with T1D was tested using hierarchical regression. The results are presented in Table 3; Fig. 1. In the first step, a regression analysis (model 1) was conducted to investigate the relationship between diabetes distress (independent variable) and self-regulatory fatigue (dependent variable) while controlling for sex, BMI, and residence. The findings revealed a significant prediction of self-regulatory fatigue by diabetes distress (*b* = 0.665, *p* < 0.001). Next, another regression analysis (model 2) was performed to explore the association between diabetes distress (independent variable) and DEBs (dependent variable), with adjustments for sex, BMI, and residence. The results demonstrated a significant total effect of diabetes distress on DEBs (*b* = 0.971, *p* < 0.001). Finally, self-regulatory fatigue was added to Model 2 as an independent variable. Both diabetes distress and self-regulatory fatigue showed significant associations with DEBs. The direct effect of diabetes distress on DEBs was estimated to be 0.477 (as shown in Fig. 1). The preceding analysis suggested that self-regulatory fatigue partly mediated the relationship between diabetes distress and DEBs. To further validate the mediating effect of self-regulatory fatigue, Model 4 of the SPSS 22.0 macro-PROCESS was employed. The biased-corrected percentile bootstrap results indicated an indirect effect of diabetes distress on DEBs, with an estimated value of 0.494, accounting for 50.88% of the overall effect. The 95% confidence interval (CI) for the indirect effect was [0.310, 0.695], as illustrated in Table 4.

**Table 3:** Hierarchical regression examining the effect of diabetes distress on self-regulatory fatigue and disordered eating behaviors among youths with type 1 diabetes

	Model 1 (Dependent variable: self-regulatory fatigue)				Model 2 (Dependent variable: disordered eating behaviors)			
	Unstandardized *b*	Standardized *β*	95% CI	*p*	Unstandardized *b*	Standardized *β*	95% CI	*p*
*Control variables*								
Male	2.452	0.170	(0.633, 4.271)	0.009	4.103	0.202	(1.662, 6.544)	0.001
Residence 1	− 0.737	− 0.052	(− 3.077, 1.603)	0.535	− 2.858	− 0.142	(− 5.999, 0.282)	0.074
Residence 2	1.359	0.085	(− 1.220, 3.938)	0.300	− 0.801	− 0.036	(− 4.262, 2.659)	0.648
BMI 1	− 0.577	− 0.027	(− 3.335, 2.181)	0.680	− 1.343	− 0.044	(− 5.043, 2.358)	0.475
BMI 2	− 1.078	− 0.053	(− 3.621, 1.466)	0.404	5.697	0.200	(2.283, 9.111)	0.001
*Independent variable*								
Diabetes distress	0.665	0.448	(0.472, 0.859)	< 0.001	0.971	0.462	(0.712, 1.231)	< 0.001
	*R*^2^ = 0.289				*R*^2^ = 0.360			
	Δ*R*^2^ = 0.289				Δ*R*^2^ = 0.360			
	* F*(6, 178) = 12.084				* F*(6, 178) = 16.703			
	*p* < 0.001				*p* < 0.001			

Note: Sex, residence, and BMI were encoded as dummy variables. The reference category for sex is female. The reference category for residence is “countryside,” where Residence 1 represents “city,” and Residence 2 represents “town.” The reference category for BMI is “normal weight,” where BMI 1 represents “underweight” and BMI 2 represents “overweight or obese"

### Testing for the moderated mediation model

**Fig. 2:**
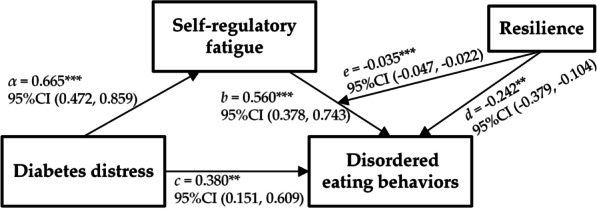
The moderated mediation model of diabetes distress, self-regulatory fatigue, resilience, and disordered eating behaviors.** α** The effect of diabetes distress on self-regulatory fatigue;** b** The effect of self-regulatory fatigue on disordered eating behaviors;** c** The direct effect of diabetes distress on disordered eating behaviors;** d** The effect of resilience on disordered eating behaviors;** e** The effect of the interaction term of resilience and self-regulatory fatigue on disordered eating behaviors ***p < 0.001; **p < 0.01. Unstandardized beta coefficients were reported

**Fig. 3:**
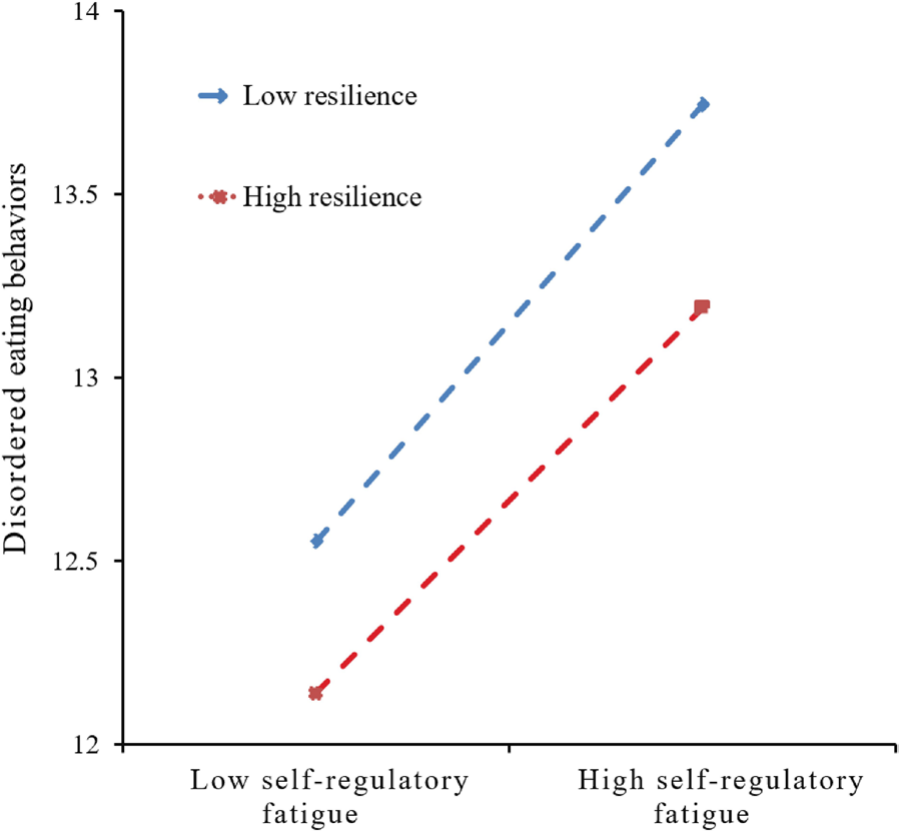
Moderating effect of resilience on the pathway from self-regulatory fatigue to disordered eating behaviors

Whether resilience buffers the impact of self-regulatory fatigue on DEBs was tested using hierarchical regressions. The results of the moderated mediation model are shown in Fig. 2. Resilience revealed its significance (*b* = − 0.147, *p* < 0.05) when sex, residence, BMI, diabetes distress and self-regulatory fatigue were controlled. Based on the previous step, a significant two-way interaction (Resilience × Self-regulatory fatigue) was added and observed in predicting DEBs (*b* = − 0.035, *p* < 0.001), indicating the moderating effect of resilience in the pathway from diabetes distress to DEBs through self-regulatory fatigue (as presented in Fig. 2). Subsequent biased-corrected percentile bootstrap results by Model 14 of the SPSS 22.0 macro-PROCESS yielded a moderated mediation index of − 0.023, with a 95% confidence interval (CI) of [− 0.035, − 0.013]. Specifically, when resilience was low (e.g., one standard deviation below the mean), self-regulatory fatigue significantly mediated the association between diabetes distress and DEBs, with a mediating index of 0.580 and a 95% CI of [0.364, 0.811]. When resilience was high (e.g., one standard deviation above the mean), the mediating effect of self-regulatory fatigue was 0.165, with a 95% CI of [0.018, 0.327] (as presented in Table 4). To gain further insights into the moderating effect of resilience, a simple slope analysis was conducted. The results showed that self-regulatory fatigue significantly influenced DEBs when resilience was low (e.g., one standard deviation below the mean; *b*_simple_ = 0.872, *p* < 0.001). Similarly, in the presence of high resilience (e.g., one standard deviation above the mean; *b*_simple_ = 0.248, *p* < 0.001), self-regulatory fatigue also predicted DEBs (as illustrated in Fig. 3).

**Table 4:** The bootstrap results of mediation and moderated mediation analysis

	Effect	SE	95%CI
*Mediation effect*			
Diabetes distress →Self-regulatory fatigue →Disordered eating behaviors	0.494	0.098	(0.310, 0.695)
*Moderated mediation effect*			
Diabetes distress →Self-regulatory fatigue →Disordered eating behaviors			
Index of moderated mediation	− 0.023	0.006	(− 0.035, − 0.013)
Low resilience	0.580	0.102	(0.364, 0.811)
High resilience	0.165	0.079	(0.018, 0.327)

Note: Effect: unstandardized regression coefficient

## Discussion

This current study is the first to determine how diabetes distress affects DEBs. As predicted, the results suggest that (1) self-regulatory fatigue acts as a mediator in the relationship between diabetes distress and DEBs, and (2) resilience moderates the connection between self-regulatory fatigue and DEBs. Moreover, the mediating effect of self-regulatory fatigue is buffered by resilience.

The findings of this study are congruent with previous research, which supports the positive association between diabetes distress and DEBs [11–13]. The general strain theory also points out that individuals may resort to maladaptive behaviors like binge eating, substance abuse, or internet addiction as a means to alleviate negative emotions or cope with distressing feelings [39].

The results of this study suggested that self-regulatory fatigue served as an intermediary between diabetes distress and DEBs, providing insights into how diabetes distress influences DEBs. According to Wagner et al., self-regulatory fatigue amplifies impulses and negative emotions while redirecting attention toward rewarding cues [19]. Moreover, neural evidence indicates that individuals with self-regulatory fatigue exhibit decreased functional connectivity with the ventromedial prefrontal cortex, a brain region that regulates emotional responses in the amygdala [19]. These findings suggest that individuals with self-regulatory fatigue struggle with self-control and regulating negative emotions. DEBs can be viewed as specific strategies for regulating the effect associated with difficulties in controlling and mentalizing emotions [40]. Hence, it is unsurprising that youth with T1D who experience self-regulatory fatigue are more likely to engage in DEBs when confronted with diabetes distress.

In this study, we found that resilience moderated the second stage of the relationship between diabetes distress and DEBs through self-regulatory fatigue. Among youth with a low level of resilience, experiencing diabetes distress increased the likelihood of engaging in DEBs through self-regulatory fatigue. However, this relationship was weakened for resilient youth. According to Rutter’s resilience development model, persons with stronger resilience can effectively use their internal strength and external environmental resources to buffer DEBs caused by self-regulatory fatigue [25]. Furthermore, evidence suggests that resilient individuals can activate specific brain structures and neural circuits to regulate emotions and exercise top-down control over emotional attention [41]. Additionally, Hood et al. conducted a one-year resilience program and observed improvements in diabetes distress [42], highlighting the potential value of resilience as a target for interventions addressing DEBs.

### Limitations and practical implications

Interpreting the present findings requires caution, given that our study has some limitations. Firstly, the cross-sectional design used in this study makes it challenging to establish causality between variables. Future research should employ longitudinal designs to validate the causal relationships proposed. Secondly, the study sample was limited to participants from China, which may restrict the generalizability of the findings to other cultural contexts. Conducting studies with diverse cultural populations would help assess the robustness of the results. Thirdly, all measures used in this study, including diabetes distress, self-regulatory fatigue, resilience, and DEBs, relied on self-report data. Despite participants not being informed in advance that their responses would be checked, the non-anonymous nature of the questionnaires may have introduced response bias. Future research should incorporate multiple data collection methods, such as face-to-face interviews and evaluations from other participants (e.g., teachers, classmates, and parents), while ensuring anonymity. Fourthly, the study was conducted during the peak of the COVID-19 pandemic in China, which might have exacerbated diabetes distress in youth with T1D and influenced the prevalence of DEBs. Lastly, the study focused only on diabetes distress, self-regulatory fatigue, and resilience as factors influencing DEBs, neglecting other physiological, psychological, and sociocultural factors contributing to the development of DEBs. Future studies should adopt a multifaceted approach to gain a more comprehensive understanding of the topic. Additionally, considering the mediating role of resilience between diabetes distress and DEBs would provide further insights and should be explored in future research.

Despite its limitations, the present study provides empirical evidence supporting the hypotheses that self-regulatory fatigue mediates the relationship between diabetes distress and DEBs, and resilience moderates the pathway from diabetes distress to self-regulatory fatigue to DEBs. These findings contribute to the existing literature on the impact of diabetes distress on DEBs and offer valuable guidance for DEBs interventions in clinical practice: (1) The study highlights the importance of including diabetes distress in healthcare providers’ DEB reduction programs; (2) Interventions targeting the reduction of self-regulatory fatigue are essential in preventing and addressing DEBs; (3) Considering the moderating role of resilience, healthcare providers should focus on fostering resilience in patients to weaken the impact of self-regulatory fatigue on DEBs. Resilience-building interventions can be incorporated into clinical care to help individuals with T1D develop coping strategies and enhance their ability to manage negative emotions and self-regulatory fatigue effectively. In clinical practice, we recommend that healthcare professionals regularly conduct psychological screenings to assess self-regulatory fatigue, diabetes distress, and resilience. These assessments can inform personalized interventions and aid in identifying individuals at higher risk of developing DEBs. Integrating these three variables into DEB reduction programs can significantly enhance intervention effectiveness.

## Conclusion

Our study explores how diabetes distress affects DEBs, although further confirmation and extensions are required. We found evidence supporting the mediating role of self-regulatory fatigue between diabetes distress and DEBs and the moderating effect of resilience on the relationship between self-regulatory fatigue and DEBs. Additionally, we observed that diabetes distress increased the likelihood of DEBs through self-regulatory fatigue, particularly among individuals with low resilience. However, this association was weakened among those with high resilience. The current study highlights the necessity of integrating resilience, self-regulatory fatigue, and diabetes distress in DEBs prevention or reduction programs.

## Data Availability

This study data is available from the corresponding author on reasonable request.

## Abbreviations

DEBs: Disordered eating behaviors
T1D: Type 1 diabetes
PAID: Problem areas in the diabetes
DSTAR: Diabetes strengths and resilience
SRF-S: Self-regulatory fatigue scale
DEPS-R: Diabetes eating problem survey-revised
SD: Standard deviation
CI: Confidence interval

## References

[1] Chiang JL, Maahs DM, Garvey KC, Hood KK, Laffel LM, Weinzimer SA (2018). Type 1 diabetes in children and adolescents: a position statement by the American Diabetes Association. Diabetes Care.

[2] Holt RIG, DeVries JH, Hess-Fischl A, Hirsch IB, Kirkman MS, Klupa T (2021). The management of type 1 diabetes in adults. A Consensus Report by the american Diabetes Association (ADA) and the European Association for the study of diabetes (EASD). Diabetes Care.

[3] Luyckx K, Verschueren M, Palmeroni N, Goethals ER, Weets I, Claes L (2019). Disturbed eating behaviors in adolescents and emerging adults with type 1 diabetes: a one-year prospective study. Diabetes Care.

[4] Rancourt D, Foster N, Bollepalli S, Fitterman-Harris HF, Powers MA, Clements M (2019). Test of the modified dual pathway model of eating disorders in individuals with type 1 diabetes. Int J Eat Disord.

[5] Wisting L, Frøisland DH, Skrivarhaug T, Dahl-Jørgensen K, Rø O (2013). Disturbed eating behavior and omission of insulin in adolescents receiving intensified insulin treatment: a nationwide population-based study. Diabetes Care.

[6] Takii M, Uchigata Y, Tokunaga S, Amemiya N, Kinukawa N, Nozaki T (2008). The duration of severe insulin omission is the factor most closely associated with the microvascular complications of type 1 diabetic females with clinical eating disorders. Int J Eat Disord.

[7] Shapiro MS (2022). Special psychosocial issues in diabetes management: diabetes distress, disordered eating, and Depression. Prim Care.

[8] Hagger V, Hendrieckx C, Cameron F, Pouwer F, Skinner TC, Speight J (2017). Cut points for identifying clinically significant diabetes distress in adolescents with type 1 diabetes using the PAID-T: results from diabetes MILES Youth-Australia. Diabetes Care.

[9] Fegan-Bohm K, Minard CG, Anderson BJ, Butler AM, Titus C, Weissberg-Benchell J (2020). Diabetes distress and HbA1c in racially/ethnically and socioeconomically diverse youth with type 1 diabetes. Pediatr Diabetes.

[10] Inverso H, LeStourgeon LM, Parmar A, Bhangui I, Hughes B, Straton E (2022). Demographic and glycemic factors linked with diabetes distress in teens with type 1 diabetes. J Pediatr Psychol.

[11] Araia E, King RM, Pouwer F, Speight J, Hendrieckx C (2020). Psychological correlates of disordered eating in youth with type 1 diabetes: results from diabetes MILES Youth-Australia. Pediatr Diabetes.

[12] Garrett CJ, Ismail K, Fonagy P (2021). Understanding developmental psychopathology in type 1 diabetes through attachment, mentalisation and diabetes distress. Clin Child Psychol Psychiatry.

[13] McClintock JM, Blackmore T, Chepulis LM, Fraser S, Paul RG (2022). The psychological profile of youth and young adults with type 1 diabetes in New Zealand. Pediatr Diabetes.

[14] Hagger MS, Wood C, Stiff C, Chatzisarantis NL (2010). Ego depletion and the strength model of self-control: a meta-analysis. Psychol Bull.

[15] Baumeister RF, Muraven M, Tice DM (2000). Ego depletion: a resource model of volition, self-regulation, and controlled processing. Soc Cogn.

[16] Baumeister RF, Vohs KD, Tice DM (2007). The strength model of self-control. Curr Dir Psychol Sci.

[17] Nes LS, Ehlers SL, Whipple MO, Vincent A, Self-Regulatory Fatigue (2017). A missing link in understanding fibromyalgia and other chronic multisymptom illnesses. Pain Pract.

[18] Eisenlohr-Moul TA, Burris JL, Evans DR (2013). Pain acceptance, psychological functioning, and self-regulatory fatigue in temporomandibular disorder. Health Psychol.

[19] Wagner DD, Altman M, Boswell RG, Kelley WM, Heatherton TF (2013). Self-regulatory depletion enhances neural responses to rewards and impairs top-down control. Psychol Sci.

[20] Ye B, Wang R, Liu M, Wang X, Yang Q (2021). Life history strategy and overeating during COVID-19 pandemic: a moderated mediation model of sense of control and coronavirus stress. J Eat Disord.

[21] Yilmaz Kafali H, Atik Altinok Y, Ozbaran B, Ozen S, Kose S, Tahillioglu A (2020). Exploring emotional dysregulation characteristics and comorbid psychiatric disorders in type 1 diabetic children with disordered eating behavior risk. J Psychosom Res.

[22] Boselie J, Vancleef LMG, Smeets T, Peters ML (2014). Increasing optimism abolishes pain-induced impairments in executive task performance. Pain.

[23] Kumfer KL. Factors and processes contributing to resilience: the resilience framework. In: Resilience and development: positive life adaptation; 1999. pp. 179–224.

[24] Kim GM, Lim JY, Kim EJ, Park SM (2019). Resilience of patients with chronic diseases: a systematic review. Health Soc Care Community.

[25] Rutter M (1999). Resilience concepts and findings: implications for family therapy. J Family Therapy.

[26] Fergerson AK, Brausch AM (2022). Resilience mediates the relationship between PTSD symptoms and disordered eating in College Women who have experienced sexual victimization. J Interpers Violence.

[27] Yang C, Zhou Y, Cao Q, Xia M, An J (2019). The relationship between self-control and self-efficacy among patients with substance use disorders: resilience and self-esteem as mediators. Front Psychiatry.

[28] McGuire BE, Morrison TG, Hermanns N, Skovlund S, Eldrup E, Gagliardino J (2010). Short-form measures of diabetes-related emotional distress: the Problem Areas in Diabetes Scale (PAID)-5 and PAID-1. Diabetologia.

[29] Nicolucci A, Kovacs Burns K, Holt RI, Comaschi M, Hermanns N, Ishii H (2013). Diabetes attitudes, wishes and needs second study (DAWN2™): cross-national benchmarking of diabetes-related psychosocial outcomes for people with diabetes. Diabet Med.

[30] Hilliard ME, Iturralde E, Weissberg-Benchell J, Hood KK (2017). The diabetes strengths and resilience measure for adolescents with type 1 diabetes (DSTAR-Teen): validation of a new, brief self-report measure. J Pediatr Psychol.

[31] Xu J, Luo D, Zhu M, Wang H, Shi Y, Ya D (2020). Translation and its psychometric characteristic of the diabetes strengths and resilience measure among chinese adolescents with type 1 diabetes. J Pediatr Nurs.

[32] Nes LS, Ehlers SL, Whipple MO, Vincent A (2013). Self-regulatory fatigue in chronic multisymptom illnesses: scale development, fatigue, and self-control. J Pain Res.

[33] Wang L, Yu Y, Tao T, Zhang J, Gao W (2018). The self-care dilemma of type 2 diabetic patients: the mechanism of self-regulation resource depletion. PLoS One.

[34] Gao Y, Shan Y, Jiang T, Cai L, Zhang F, Jiang X (2021). Dietary adherence, self-regulatory fatigue and trait self-control among Chinese patients with peritoneal dialysis: a cross-sectional study. Patient Prefer Adherence.

[35] Antisdel J, Laffel LMB, Anderson BJ (2001). Improved detection of eating problems in women with type 1 diabetes using a newly developed survey. Diabetes.

[36] Markowitz JT, Butler DA, Volkening LK, Antisdel JE, Anderson BJ, Laffel LM (2010). Brief screening tool for disordered eating in diabetes: internal consistency and external validity in a contemporary sample of pediatric patients with type 1 diabetes. Diabetes Care.

[37] Lv W, Zhong Q, Guo J, Luo J, Dixon J, Whittemore R (2021). Instrument context relevance evaluation, translation, and psychometric testing of the diabetes eating problem survey-revised (DEPS-R) among people with type 1 diabetes in China. Int J Environ Res Public Health.

[38] Hayes AF, Rockwood NJ (2017). Regression-based statistical mediation and moderation analysis in clinical research: observations, recommendations, and implementation. Behav Res Ther.

[39] Aseltine RH, Gore S, Gordon J (2000). Life stress, anger and anxiety, and delinquency: an empirical test of general strain theory. J Health Soc Behav.

[40] Biberdzic M, Tang J, Tan J (2021). Beyond difficulties in self-regulation: the role of identity integration and personality functioning in young women with disordered eating behaviours. J Eat Disord.

[41] van der Werff SJ, van den Berg SM, Pannekoek JN, Elzinga BM, van der Wee NJ (2013). Neuroimaging resilience to stress: a review. Front Behav Neurosci.

[42] Hood KK, Iturralde E, Rausch J, Weissberg-Benchell J (2018). Preventing diabetes distress in adolescents with type 1 diabetes: results 1 year after participation in the STePS Program. Diabetes Care.

